# Rifampicin–Liposomes for *Mycobacterium abscessus* Infection Treatment: Intracellular Uptake and Antibacterial Activity Evaluation

**DOI:** 10.3390/pharmaceutics13071070

**Published:** 2021-07-13

**Authors:** Federica Rinaldi, Patrizia Nadia Hanieh, Simona Sennato, Federica De Santis, Jacopo Forte, Maurizio Fraziano, Stefano Casciardi, Carlotta Marianecci, Federico Bordi, Maria Carafa

**Affiliations:** 1 Dipartimento di Chimica e Tecnologie del Farmaco, Sapienza Università di Roma-Piazzale Aldo Moro 5, 00185 Rome, Italy; federica.rinaldi@uniroma1.it (F.R.); patrizianadia.hanieh@uniroma1.it (P.N.H.); jacopo.forte@uniroma1.it (J.F.); maria.carafa@uniroma1.it (M.C.); 2 Istituto dei Sistemi Complessi (ISC)-CNR, sede “Sapienza” and Dipartimento di Fisica, Sapienza Università di Roma, 00185 Rome, Italy; federico.bordi@roma1.infn.it; 3 Dipartimento di Biologia, Università di Roma “Tor Vergata” Via della Ricerca Scientifica, 00133 Rome, Italy; federica.desa92@gmail.com (F.D.S.); fraziano@bio.uniroma2.it (M.F.); 4 Department of Occupational and Environmental Medicine, Epidemiology and Hygiene, National Institute for Insurance against Accidents at Work (INAIL), Monteporzio Catone, 00144 Rome, Italy; s.casciardi@inail.it

**Keywords:** liposomes, rifampicin, *Mycobacterium abscessus*, antibiotic resistance

## Abstract

Treatment of pulmonary infections caused by *Mycobacterium abscessus* are extremely difficult to treat, as this species is naturally resistant to many common antibiotics. Liposomes are vesicular nanocarriers suitable for hydrophilic and lipophilic drug loading, able to deliver drugs to the target site, and successfully used in different pharmaceutical applications. Moreover, liposomes are biocompatible, biodegradable and nontoxic vesicles and nebulized liposomes are efficient in targeting antibacterial agents to macrophages. The present aim was to formulate rifampicin-loaded liposomes (RIF–Lipo) for lung delivery, in order to increase the local concentration of the antibiotic. Unilamellar liposomal vesicles composed of anionic DPPG mixed with HSPC for rifampicin delivery were designed, prepared, and characterized. Samples were prepared by using the thin-film hydration method. RIF–Lipo and unloaded liposomes were characterized in terms of size, ζ-potential, bilayer features, stability and in different biological media. Rifampicin’s entrapment efficiency and release were also evaluated. Finally, biological activity of RIF-loaded liposomes in Mycobacterium abscessus-infected macrophages was investigated. The results show that RIF-lipo induce a significantly better reduction of intracellular Mycobacterium abscessus viability than the treatment with free drug. Liposome formulation of rifampicin may represent a valuable strategy to enhance the biological activity of the drug against intracellular mycobacteria.

## 1. Introduction

*Mycobacterium abscessus* (*Mabs*) is an emerging human pathogen belonging to non-tuberculous mycobacteria which is responsible for severe pulmonary infections chiefly in immunocompromised individuals [1]. Nevertheless, there is evidence of infections in immunocompetent individuals, and, furthermore, it is able to cause extra-pulmonary infections affecting mainly the skin but also the eyes, soft tissue, bone, joint, and central nervous system [2]. Due to the severity of *Mabs* infection in association with the intrinsic capability of this pathogen of being highly drug-resistant, *Mabs* is considered one of the most worrying mycobacteria. The severe antimicrobial resistance associated with unfavorable physicochemical properties and toxicity of the many existing antimicrobial drugs make the treatment of *Mabs* infections particularly challenging [3]. The waxy coating of *Mabs* cell wall, mainly composed of mycolic acids, acts as an extraordinary barrier conferring resistance to dehydration, a low permeability to hydrophobic antibiotics, and a high ability of surviving [3]. Last, its intracellular localization offers protection not only from the host defenses and the immune responses but also from the action of conventional antimicrobial agents, so that the effective treatment of infections remains a major pharmaceutical challenge [4].

Liposomes have been tested with various drugs, and liposome-formulated drugs are now in clinical use for cancer and systemic or local infections, as well as for the treatment of chronic respiratory conditions [5]. Antibiotics encapsulated in liposomes have been shown to be effective both in tuberculous [6] and in non-tuberculous mycobacteria strains [7]. The currently available guidelines state that there are no specific treatments, treatment combinations, or therapeutic regimens with proven efficacy [8]. In addition, the current available or developing treatment for *Mabs* infection remains weak and not entirely successful; thus, there is an urgent need to discover more effective drugs for a reliable and faster treatment of *Mabs* lung disease [9].

Rifampicin (RIF) is a key antibiotic in the frontline treatment of Tuberculosis infections and shows an exceptional bactericidal activity against intra-macrophage and (drug tolerant) nonreplicating bacteria [10]. However, it is not included in the clinical treatment of *Mabs* lung disease, since *Mabs* is intrinsically resistant to rifampicin. The recent discovery that rifabutin, an analogue of rifampicin, is active against *Mabs* [11] has stimulated different strategies to reposition rifampicin for the treatment of this difficult non-tuberculous mycobacteria lung disease. In this context, RIF encapsulation in a proper nanocarrier could represent a viable strategy to increase its local concentration and overcome mycobacterial drug inactivation, offering, at the same time, the possibility to increase its stability and water solubility and have a favorable impact on *Mabs* infection [12].

The aim of this study was to define a novel liposomal formulation entrapping rifampicin with high antibacterial activity towards *Mabs*. We have considered liposomal vesicles composed of an equimolar mixture of the zwitterionic hydrogenated phosphatidylcholine from soybean (HSPC) and the anionic 1,2-Dipalmitoyl-sn-glycero-3-phosphorylglycerol sodium salt (DPPG) lipids that have not been investigated so far. HSPC is already employed in several approved liposomal drugs [13]. The inclusion of the anionic lipid has been motivated by at least two reasons. First, it was shown that anionic particles are more likely to be phagocytosed by macrophages due to resembling negatively charged bacteria cells [14]. For this reason, anionic liposomes may be more beneficial for intracellular infections that reside inside phagocytic cell types. This can give reason of the threefold increase in the uptake of rifampicin-loaded anionic liposomes in lungs macrophages, compared with the free antibiotic and neutral liposomes, observed in the earlier investigation of Vyas et al. [15]. Furthermore, the addition of DPPG gives the further possibility of exploiting polymeric chitosan coatings to confer mucoadhesion properties and improve mechanical properties, which is relevant for pulmonary delivery [16].

Here we performed deep physicochemical characterizations in terms of the hydrodynamic size, ζ-potential, morphology, vesicle bilayer characteristics, and physical and biological effectiveness. We showed that the used drug–lipid combination, which has not been previously described in the literature, to the best of our knowledge, possesses significant benefits in term of drug solubility and degradation protection, and antibacterial effect on *Mabs* infection. For all of these relevant properties, HSPC–DPPG liposomes loading rifampicin appear a valid candidate to treat *Mabs* pulmonary infections.

## 2. Materials and Methods

### 2.1. Materials

Hydrogenated phosphatidylcholine from soybean (HSPC) with molecular weight Mw = 790 g/mol and anionic 1,2-Dipalmitoyl-sn-glycero-3-phosphorylglycerol sodium salt (DPPG) with molecular weight Mw = 745 g/mol were a kind gift from LIPOID GmbH (Ludwigshafen, Germany). The typical fatty acid composition (expressed in % of total fatty acids) of HSPC is: Palmitic acid: (5.0–20.0%) and Stearic acid (80.0–95.0%). Sodium calcein, rifampicin (RIF) (nominal purity >97%), Sephadex G75, diphenylhexatriene (DPH), calcein, and Hepes salt [N-(2-hydroxyethyl)piperazine-N′-(2-ethanesulfonic acid)] were purchased by Sigma-Aldrich (St. Louis, MO, USA). This drug has a LogP = 3719; thus, it is associated to a very low water solubility [H2O: soluble 2.5 mg/mL at 25 °C (pH 7.3) chloroform: soluble 50 mg/mL [17].

All other products and reagents were of analytical grade.

### 2.2. Preparation of Liposomes and Rifampicin-Loaded Liposomes

Liposomes and RIF-loaded liposomes were prepared by Thin-Layer Evaporation technique as previously reported [18]. Sample composition is reported in Table 1. Briefly, 5 mg of HSPC and DPPG lipids were dissolved in a round-bottom flask with a chloroform/methanol mixture (3:1 *v/v*). Organic solvents were evaporated for one hour at 60 °C by using a Rotavapor^®^ R-210 (Büchi-Italia S.r.l., Assago (MI), Italy) under vacuum to obtain a thin lipid film. Residual organic solvent was removed under vacuum overnight at room temperature, using a T51 glass oven dryer (Büchi-Italia S.r.l., Assago (MI), Italy). The lipid film was then hydrated in 5 mL Hepes buffer (pH = 7.4 0.01 M) or calcein solution (0.01 M) [18] by using a vortex mixer and a water bath (60 ± 1 °C for 3 min). Tip-sonication under nitrogen flux (amplitude 16%, temperature 4 °C, time minutes, pulse on 0.8 and pulse off 0.6) was used to reduce the size of multilamellar vesicles.

For RIF-loaded liposomes, 5 mg of drug has been dissolved with lipids and the same preparation protocol has been used. Centrifugation at 18,000 rpm and 4 °C for 30 min has been used to remove the un-entrapped RIF remaining in the precipitate.

For calcein RIF-loaded liposomes, centrifugation at 18,000 rpm and 4 °C for 30 min has been used and the untrapped calcein in the vesicles was removed by dialysis against DI water for 10 h, using a dialysis bag with molecular weight cutoff (MWCO) of 1000 and the external medium (water) was removed 3 times, at 2, 4, and 6 h.

After preparation, all samples were stored at 4 °C until their use in different experiments.

### 2.3. Rifampicin Entrapment Efficiency (EE%)

Entrapment efficiency (*EE*) of rifampicin in liposomal vesicles was evaluated by UV–vis spectrometer (Lambda 25, PerkinElmer, Waltham, MA, USA). Purified liposomes were diluted ten times in Ethanol:Hepes 1:1 vol:vol and absorbance of RIF at λ = 465 nm was measured by using Ethanol:Hepes 1:1 solution as a reference [19].

The *EE* was calculated as follows:

(1) $$EE\left(\%\right)=\frac{Entrapped\;drug\;\left(mg\right)}{Total\;drug\;used\;\left(mg\right)}\times 100$$

The results are shown as the average of three different batches ± standard deviation.

The stability of RIF entrapment in liposomes was also controlled at two different temperatures, 4 °C and room temperature, and after 1, 30, 60, and 90 days.

### 2.4. In Vitro Release Studies

In vitro release experiments were used to test drug release from liposomes. Experiments were carried out by using dialysis tubes (molecular weight cutoff 8000 and 5.5 cm^2^ diffusing area) at 37 °C in Hepes buffer (10 mM, pH 7.4) in a release medium Ethanol:Hepes 1:1, which was gently magnetically stirred during the experiment.

The sample was diluted 1:10 with the medium to obtain the highest absorbance. RIF concentration in the release medium was measured by using the UV spectrophotometer (Perkin-Elmer, lambda 3a, UV–Vis spectrometer), as described above, at different time points over 1–24 h. Aliquots of 1 mL were withdrawn from the solution at different time points to perform UV analysis and then reinserted back in the external medium. All samples were analyzed immediately after sampling. Reported values represent the mean values over three repeated independent experiments and errors are the standard deviation.

### 2.5. Size and ζ-Potential Measurements

Liposome size and ζ-potential were measured by using a Malvern NanoZetaSizer apparatus (Malvern Instruments, Worcestershire, UK), equipped with a 5 mW HeNe laser (λ = 632.8 nm). In Dynamic light scattering (DLS) measurements the scattered intensity was detected at a 90-degree angle and the normalized intensity autocorrelation functions were calculated by a logarithmic digital correlator and analyzed by using the cumulant method to get the values of the hydrodynamic diameter and the polydispersity index (PDI) [20]. For the determination of ζ-potential, electrophoretic mobility μ was measured by the dielectrophoretic light scattering (DELS) by the same apparatus used for DLS. The measured electrophoretic mobility µ was converted into the ζ-potential, using the Smoluchowski relation ζ = µ η/ε, where η and ε are the viscosity and the permittivity of the solvent phase, respectively [21].

### 2.6. Morphological Investigation

Transmission Electron Microscopy (TEM) and Atomic Force Microscopy (AFM) were used to visualize the morphology of the liposomes as well as their interaction with culture medium. Samples for TEM were prepared on copper grids covered with a carbon film. The vesicle dispersions were pipetted onto the grids and stained with 1% phosphotungstic acid (pH adjusted to 7.4) as performed in previous investigations [22]. Measurements were carried out by means of a FEI TECNAI 12 G2 Twin (FEI Company, Hillsboro, OR, USA), operating at 120 kV and equipped with an electron energy filter (Gatan image filter) and a slow-scan charge-coupled device camera (Gatan multiscan).

Atomic Force Microscopy (AFM) measurements were performed with a Dimension Icon (Bruker AXS, Billerica, MA, USA) instrument in air at room temperature and under ambient conditions. We used Tapping mode and RTESP-300 (Brucker) probes characterized by a sharp silicon tip (nominal radius of curvature 10 nm). Liposomal suspension was diluted 50× with Hepes buffer and then incubated on freshly cleaved mica for 10 min. After liposomes adsorption, the substrate surface was washed three times with Milli-Q water to remove the non-adsorbed particles. AFM imaging has been performed in air and at room conditions. Images were analyzed by using Gwyddion free software. Height sensor data were presented as raw data, except for flattening and background subtraction.

### 2.7. Physicochemical Stability

Liposomal formulations entrapping RIF were stored at 4 and 25 °C for a period of 90 days and stability studies were carried out by DLS and DELS and UV–Vis. Samples from each batch of empty liposomes and RIF-loaded liposomes were withdrawn at fixed time intervals (1, 30, 60, and 90 days) and the mean of hydrodynamic diameter and PDI, ζ-potential and drug entrapment efficiency (*EE*%) were determined as previously described. The stability of RIF-free and RIF-loaded liposomes was also controlled at two different temperatures, 4 °C and room temperature, and after 1, 30, 60, and 90 days.

### 2.8. Bilayer Characterization by DPH Fluorescence Anisotropy

Bilayer characterization has been carried out on liposomes and RIF-liposomes by measuring the DPH fluorescence anisotropy, which is a parameter interpreted as a membrane microviscosity (viscosity in the bilayer interior) or fluidity [23]. DPH-loaded liposomes were prepared by co-dissolution in the mixture of organic solvents of lipids and probe (2 × 10^−4^ M), then the same preparation method described for empty liposomes in 2.2 was followed. DPH-liposome solution was filtered through cellulose filter of 450 nm cutoff, and its fluorescent measurements were performed with excitation λ_exc_ = 350 nm and detecting the fluorescence intensity at λ_em_ = 428 nm, using luminescence spectrometer (LS5013, PerkinElmer, Waltham, MA, USA) [24]. The fluorescence anisotropy (r) was determined by using Equation (2):

(2) $$Fluorescence\;anisotropy\;\left(r\right)=\frac{\left(I_{vv}-I_{vh}\right)\times G}{(I_{vv}+2I_{vh})\times G}$$

where I_VV_, I_VH_, I_HV_, and I_HH_ are fluorescent intensities, and subscript V (vertical) and H (horizontal) represent the orientation of polarized light and *G* = I_HV_/I_HH_ factor is the ratio of sensitivity of detection system for vertically and horizontally polarized light.

### 2.9. Biological Evaluation

#### 2.9.1. Bacterial Strains

*Mycobacterium abscessus* was purchased by American Type Culture Collection ATCC19977 Bacteria were stored at −80 °C in Middlebrook 7H9 (Difco, Detroit, MI, USA) broth supplemented with 10% ADC (albumin, dextrose and catalase), 0.05% Tween 80 and 30% glycerol (Sigma-Aldrich, St. Louis, MO, USA).

The single colony was collected by striking on Middlebrook 7H10 (Difco) supplemented with 10% OADC (oleic acid, albumin, dextrose and catalase) and then was suspended in 15 mL of 7H9 broth supplemented with 10% ADC and 0.05% Tween 80. Bacteria were grown in Erlenmeyer flask at 37 °C under stirring for 48 h and their growth was monitored by measuring the optical density at the wavelength of 600 nm by Varioskan LUX (ThermoFisher Scientific, Waltham, MA, USA).

#### 2.9.2. Cell Line

Human pro-monocytic THP-1 leukemia cell line was supplied by European Collection of Cell Culture. THP-1 was cultured at 37 °C with 5% CO_2_ in RPMI 1640 (Lonza, Basel, Switzerland) supplemented with 10% fetal bovine serum (Euroclone, Milan, Italy), 2 mM L-Glutamine (Lonza), 1 mM nonessential amino acids (Lonza), 1 mM sodium pyruvate (Lonza) and 5 μg/mL gentamycin (Lonza). For the experiments, cells were seeded in 24-well plates at the concentration of 5 × 10^5^ cells/mL or in 96-well plates at the concentration of 2 × 10^5^ cells/200 µL for 72 h in the presence of 20 ng/mL Phorbol 12-Myristate 13-Acetate (PMA, Sigma-Aldrich), getting differentiated THP-1 (dTHP-1).

#### 2.9.3. *Mabs* Bacteria Infection

First, dTHP-1 cells (5 × 10^5^ cells/mL) were infected for 3 h with *Mabs* at the multiplicity of infection (MOI) of 10 in the medium without gentamycin at 37 °C with 5% CO_2_. Afterwards the extracellular mycobacteria were killed by 1 h of incubation with 250 µg/mL Amikacin (Sigma-Aldrich) and cells were treated for 18 h with empty liposomes, RIF-loaded liposomes (RIF–Lipo), and free RIF at different concentrations, as indicated in figure legends. Finally, intracellular bacterial growth was assessed by Colony-forming unit (CFU) assay; therefore, cells were lysed with 1% deoxycholate (Sigma-Aldrich), samples diluted in PBS–Tween 80 (0.01%) and CFU quantified by plating bacilli in triplicate on 7H10 supplemented with OADC.

#### 2.9.4. Direct Effect of Rifampicin on *Mabs*

First, the effect of the administration of RIF on the growth of *Mycobacterium abscessus* has been evaluated. *Mabs* was seeded in 24-well plates (5 × 10^6^/well, to mimic the amount of bacilli used for the infection of dTHP-1), incubated for 3 h and then stimulated with different concentrations of free RIF for 18 h. Finally, samples were diluted in PBS–Tween 80 (0.01%) and CFU quantified by plating bacilli in triplicate on 7H10 supplemented with OADC.

#### 2.9.5. Stability of Liposomes in Culture Medium

As preliminary biological evaluation, the in vitro stability of liposomes in the presence of THP-1 culture medium has been carried out. RIF–liposomes, as well as empty liposomes, were diluted in culture medium to obtain a final concentration of 45%. The average size, polydispersity index, and ζ-potential were evaluated by means of DLS maintaining samples at 37 °C and performing measurements at different time points (8, 24, 28, and 72 h).

#### 2.9.6. Effect of Rifampicin and Rifampicin-Loaded Liposomes on dTHP-1

To determine the effect of RIF and RIF-loaded liposomes on infection cell model, dTHP-1 (2 × 10^5^ cells/200 µL) were stimulated with empty liposomes and with 96 µM of either free or encapsulated RIF for 18 h. Thereafter, MTT assay (Molecular Probe) was performed according to the manufacturer’s instructions and as negative control dTHP-1 were treated with 0.1% saponin at 37 °C for 30 min.

#### 2.9.7. Uptake of Liposomes in dTHP-1 Cells

First, dTHP-1 cells (5 × 10^5^ cells/mL) were stimulated for 18 h with empty liposomes and RIF–liposomes, containing or nor calcein, which is converted to a green-fluorescent calcein within live cells. The internalization was analyzed by a flow cytometer FACSCalibur (Becton Dickinson, Franklin Lakes, NJ, USA).

### 2.10. Statistical Analysis

Results of physicochemical and biological characterization are expressed as the mean of three independent experiments ± standard deviation. The statistical analysis of biological data was performed by using Student’s *t*-test. The *p*-values lower than 0.05 were considered statistically significant.

## 3. Results and Discussion

### 3.1. Characterization of Liposomes

#### 3.1.1. Physicochemical Features of Liposomal Formulations

HSPC–DPPG liposomal samples were characterized in terms of their hydrodynamic size, ζ-Potential, PDI, RIF entrapment efficiency, and anisotropy (Table 2). First, we note that the entrapment efficiency (*EE*%) of RIF, as evaluated by UV analysis, is almost 100%. DLS analyses show that RIF-loaded liposomes and the empty ones possess almost the same size. For both samples, the hydrodynamic diameter is less than 130 nm and the PDI for both samples is compatible with a monodisperse population. It is noteworthy to observe that the size of RIF-loaded liposomes and empty ones spans in the same range. This feature must be taken into account in order to obtain the same in vitro behavior of the carriers. In fact, the size of liposomal carrier affects cell internalization and has a direct impact on its efficacy or toxicity [25]. Due to the presence of the anionic DPPG, the ζ-potential of all samples is quite negative (−55.4/−41.7 mV), this assures a good colloidal stability due to the presence of electrostatic repulsion that prevent aggregation and subsequent precipitation [26,27].

As reported for pure DPPG, DPSC and DPPC liposomes in the gel state at 25 °C, mixed HSPC–DPPG liposomes are characterized by high values of anisotropy that are indicative of a rigid lipid bilayer [28]. The encapsulation of RIF in HSPC–DPPG liposomes causes a slight decrease of anisotropy, similarly to what observed in pure DMPG anionic liposomal formulations [29]. This is indicative of a small perturbation of the bilayer where the drug is deeply inserted, on the basis of its solubility properties and of the reported partition coefficient [30].

All the results show that RIF has a high degree of incorporation in HSPC–DPPG liposomes and its presence does not induce significant variation to the vesicular structure. The large encapsulated amount of this bulky drug can be due to the peculiar lipid composition and by the electrostatic/hydrophobic characteristics of liposomes. As shown by a previous DSC investigation of some of us [24], mixtures of anionic DPPG and zwitterionic HSPC possess an ideal mixing behavior and the bilayer with equimolar composition is characterized by a high degree of lipid heterogeneity. Furthermore, the small length mismatch between the hydrocarbon chains of DPPG and HSPC can introduce defects in lipid packing at the level of the hydrophobic region where RIF can find its optimal accommodation. A modification of lipid order associated the presence of drug–lipid interaction has been observed by DSC investigation of RIF–DPPC lipid membranes [31]. This aspect may deserve further investigation to shed light on the interplay between lipid–lipid and drug–lipid interactions that, in addition to the hydrophilic–lipophilic balance, can also include an electrostatic contribution due the partial ionization of drug, that is present at 40% in its anionic form at neutral pH [30].

#### 3.1.2. Physicochemical Stability

**Figure 1:**
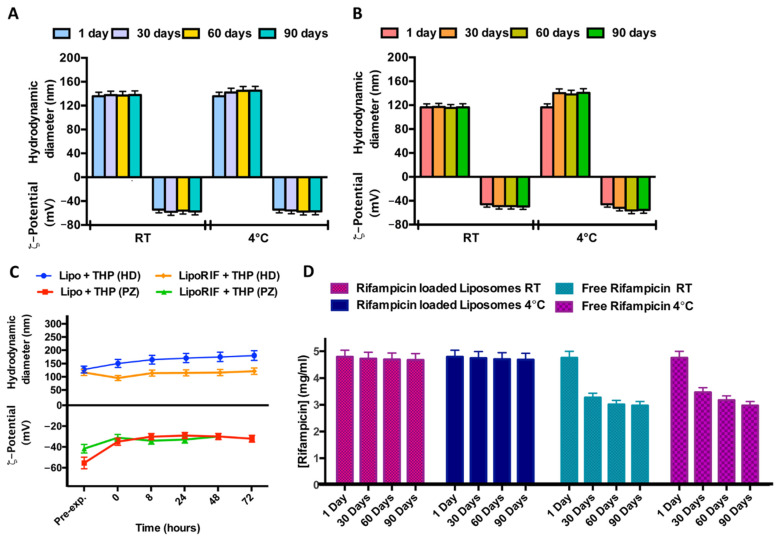
Results of investigation on physicochemical stability over time. Effect of storage temperature (room temperature (RT) and 4 °C) on hydrodynamic diameter and ζ-potential of (**A**) empty liposomes and (**B**) RIF-loaded liposomes. (**C**) Effect of THP culture medium at different incubation times on hydrodynamic diameter and ζ-potential of empty liposomes (Lipo) and RIF-loaded liposomes (RIF–Lipo). Pre-exp. values refer to liposomes before incubation with THP culture medium. (**D**) Stability studies over time at two different storage temperatures for free RIF and RIF loaded into liposomes.

In order to study the stability over time of liposomal formulations, samples were stored at two different temperatures (room temperature and 4 °C) for a period of 90 days. No significant variations of hydrodynamic diameter or ζ-potential were detected during the experiment, for both empty and RIF-loaded liposomes, so it is possible to affirm that both samples are stable at this experimental condition probably due to the quite negative ζ-potential values able to prevent aggregation or precipitation phenomena (Figure 1A,B).

Stability of empty and RIF-loaded liposomes was also evaluated at 37 °C in THP culture medium. (Figure 1C). No significant alterations in dimensions and ζ-potential within 72 h were observed so it is possible to conclude that loaded and empty liposomes are stable in this culture medium.

In order to evaluate RIF stability in terms of decomposition/degradation [32], RIF free and RIF-loaded in liposomes was analyzed by UV analysis. The UV spectra were recorded immediately after sample preparation and after 30 and 90 days at two different temperatures (room temperature and 4 °C) and the RIF concentration values were reported in Figure 1D. RIF concentration amount remains constant during time, only when it is loaded inside liposomes (RIF-loaded liposomes). It can be concluded that rifampicin inclusion in liposomes increases its stability, with respect to free drug dissolved in Hepes solution.

In the end, all the physicochemical data indicate that it is possible to obtain stable liposomal formulation to entrap RIF for antibacterial therapy.

#### 3.1.3. Morphological Investigation

**Figure 2:**
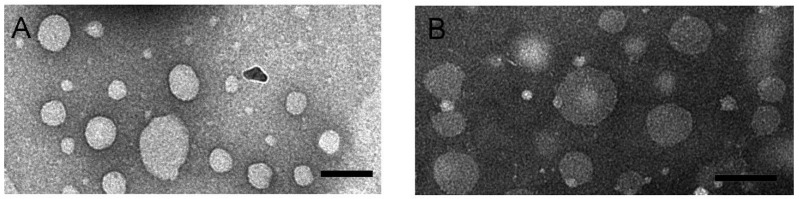
TEM images obtained by PTA staining of RIF-loaded HSPC–DPPG liposomes dissolved in Hepes buffer (**A**) and in THP culture medium (**B**). Bar represents 100 nm.

Morphology of RIF-loaded HSPC–DPPG liposomes have been studied by TEM and AFM, the two techniques giving complementary information about the liposome structure. TEM images of RIF–liposomes in Hepes solution and in THP culture medium have been shown in Figure 2A,B, respectively. Liposomes appear as non-coalescing spherical unilamellar vesicles with a not so homogeneous size distribution due to unavoidable dehydration and flattening effect [33]. The presence of THP culture medium does not alter the unilamellar nature of the liposomal vesicles, as expected on the basis of the results of stability studies.

**Figure 3:**
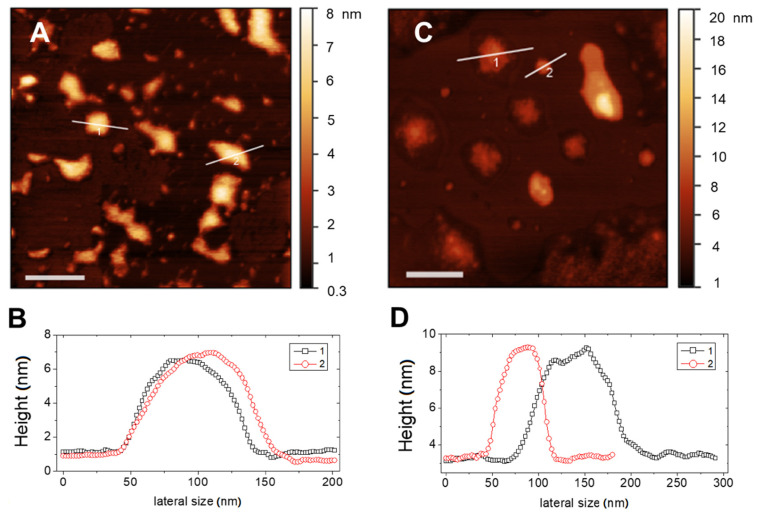
AFM images (top panels) and corresponding height profiles evaluated on the marked section (bottom panels) of RIF-loaded liposomes in different media: (**A**,**B**) Hepes and (**C**,**D**) THP culture medium. Bars represent 200 nm.

Typical AFM topographical images of RIF-loaded liposomes in Hepes and in THP culture medium are shown in Figure 3A–C, respectively. In both media, liposomes appear as large flattened spots with an average height around 6 nm and a height-to-diameter ratio of the order of 0.06, as it can be deduced by analysis of the height profiles traced on the vesicles, as those shown in Figure 3B,C. This indicates that liposomes were not attached as hemispheres but adsorbed as flat objects due to the favorable interaction with the hydrophilic mica, which overcomes the bending rigidity of the bilayer favoring vesicle opening and rupture [34]. This is not surprising, since according to prevalent theories [35,36], one would expect that rather big liposomes to adsorb and flatten to a pancake-like structure, followed by rupture to end up as single-bilayer disks by spreading.

Adsorbed liposomes appear well distinct, nevertheless they may suffer from a large deformation on the support and also of occasional liposome-to-liposome fusion events occurring between surface-adsorbed vesicles, which result in a heterogeneous size distribution. More relevant, the observed value of height of the adsorbed liposomal structures is indicative of a single lipid bilayer for both media. This confirms the unilamellar nature of the lipid vesicles and the absence of restructuring due to the presence of drug.

#### 3.1.4. Rifampicin Release Profile

**Figure 4:**
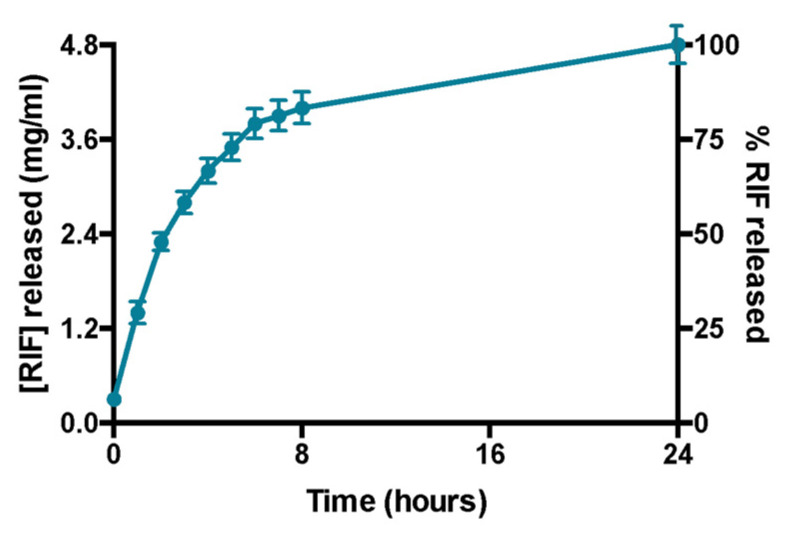
Profile of release of rifampicin over 24 h. Data were obtained as the mean of three independent experiments.

Rifampicin release experiments confirm that the amount of the drug encapsulated in the formulation is entirely released within 24 h (Figure 4). This was also demonstrated by the absence of liposomes in the acceptor compartment and by the presence of empty liposomes in donor compartment after DLS analysis (data not shown). Moreover, DLS analysis confirms the liposomes integrity in terms of size and ζ-potential in the donor compartment at the end of the experiment (data not shown).

### 3.2. Biological Evaluation of RIF-Loaded Liposomes

**Figure 5:**
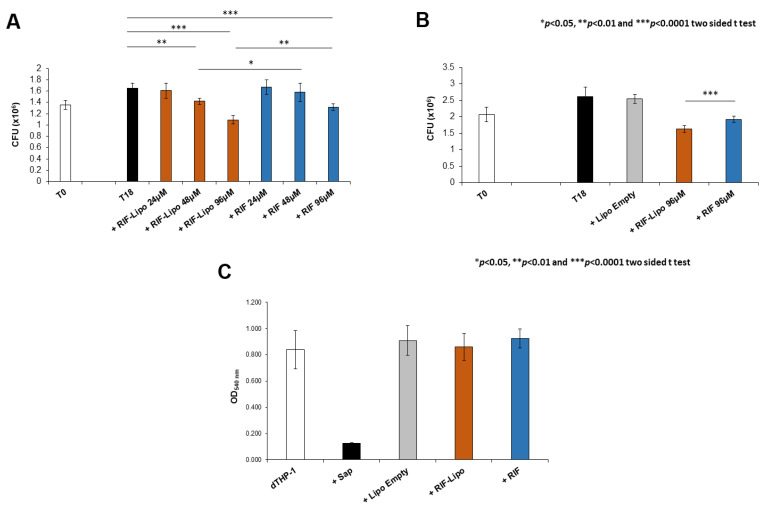
Intracellular mycobacterial killing is improved by rifampicin-loaded liposomes with no cytotoxicity effect. dTHP-1 (5 × 10^5^ cells/mL) were infected with *M. abscessus* (MOI 10) for 3 h and the extracellular bacilli were killed with 250 µg/mL Amikacin for 1 h. (**A**) Cells were treated for 18 h with RIF-loaded liposomes (RIF–Lipo) and free drug (RIF) at different concentrations and bacterial growth was quantified by CFU assay. Results are shown as mean ± standard deviation (SD) of CFU values obtained from triplicate cultures and are representative of three independent experiments. (**B**) Cells were treated with unloaded liposomes (Lipo Empty), 96 µM rifampicin-loaded liposomes (RIF–Lipo) and 96 µM free drug (RIF) for 18 h, and bacterial growth was assessed by CFU assay. Results are shown as mean ± SD of CFU values obtained from triplicate cultures and are representative of three independent experiments. * *p* < 0.05, ** *p* < 0.01 and *** *p* < 0.0001 by two sided Student’s *t*-test. (**C**) Liposome formulations and rifampicin do not exert cytotoxicity on macrophages. dTHP-1 (2 × 10^5^ cells/200 µL) were stimulated with unloaded liposomes (Lipo Empty), 96 µM rifampicin-loaded liposome (RIF–Lipo) and 96 µM free rifampicin (RIF) for 18 h and then were subjected to MTT Assay. Cells were treated with 0.1% saponin at 37 °C for 30 min as negative control (Sap). Results are shown as mean ± standard deviation (SD) of OD_540nm_ values performed in triplicate and are representative of three independent experiments.

Antimicrobial activity of RIF-loaded liposomes was evaluated in the context of *Mycobacterium abscessus* infections. Since *Mabs* is an opportunistic pathogen naturally resistant to many common antibiotics, we firstly analyzed the effect of this drug on the viability of pathogen. *Mabs* was exposed to a crescent concentration of antibiotic (from 6 to 192 µM) and bacterial growth was evaluated after 18 h by means of CFU assay. The presence of RIF promotes a significant reduction of pathogen viability only at the highest concentrations (Supplementary Materials Figure S1). On the basis of these results, we selected three concentrations to compare the effect of free antibiotic and RIF-loaded liposomes on an in vitro model of macrophage infection. In this context, *Mabs* infected macrophages were untreated or either treated with 24, 48, and 96 µM of RIF encapsulated into liposomes or with 24, 48, and 96 µM of free drug and finally intracellular mycobacterial viability was analyzed after 18 h of treatment. Results shown in Figure 5A indicate that both RIF-loaded liposomes and free RIF are able to reduce intracellular mycobacterial viability although to a significantly greater extent when encapsulated into liposomes. Moreover, this effect was mediated by the presence of antibiotic and not by liposome component, as unloaded liposomes do not influence microbial viability, excluding their involvement in the intracellular mycobacterial killing (Figure 5B). Furthermore, the selected treatments do not exert any toxic effect on macrophages (Figure 5C), excluding the possibility of a cell stress mediated antimicrobial effect [37].

**Figure 6:**
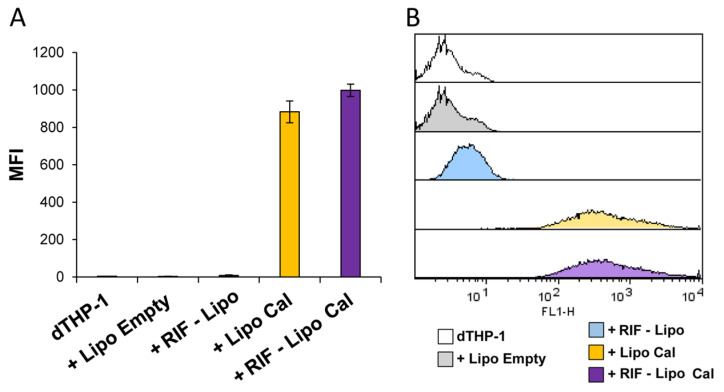
Liposome internalization analysis within macrophages. dTHP-1 (5 × 10^5^ cells/mL) were stimulated for 18 h with empty liposomes (Lipo Empty) or with RIF-loaded liposomes (RIF–Lipo), containing or not calcein (Cal). Cells were collected and liposome uptake was analyzed by flow cytometry. Results are shown as mean ± SD of mean fluorescence intensity (MFI) values obtained from three independent experiments (**A**) of which representative flow cytometry histograms are reported (**B**).

Finally, to assess the capability of RIF-loaded liposomes to be internalized in macrophages, and hence to be active against intracellular bacteria, RIF-loaded and unloaded liposomes, containing or not calcein, were prepared, dialyzed to remove free rifampicin and calcein, and used to stimulate dTHP-1 cells. Figure 6 shows that cells exposed to dialyzed RIF-loaded and unloaded liposome formulations show a similar pattern of fluorescence (Figure 6A), indicating that both liposome formulations were efficiently internalized by macrophages (Figure 6B).

## 4. Conclusions

Conventional treatment of some pulmonary infections results in different disadvantages in terms of drug-resistance, side effects, and patient’s noncompliance, due to long therapeutic treatment. In order to overcome these drawbacks, the encapsulation of rifampicin in liposomes can represent a promising strategy. A novel liposomal formulation entrapping rifampicin and composed by HSPC–DPPG was prepared and deeply characterized. Results obtained by physical–chemical characterization and by in vitro functional assays demonstrate that RIF-loaded liposomes may be good candidates for the treatment of pulmonary infections.

In particular, DLS studies confirm the liposomes nanosize and stability (in Hepes buffer and in THP culture medium). Moreover, RIF–liposomes show a good drug entrapment efficiency (close to 100%) and a suitable RIF release profile, which can be correlated to a sustained release property. This is particularly relevant in reducing the dosing frequency and the enhanced patients’ compliances. Altogether, we can conclude that HSPC–DPPG liposomes not only ensure the biological activity of RIF, but may improve it by optimizing its intramacrophage bioavailability, with a potential benefit in the treatment of the deep lung of infected patients.

## Figures and Tables

**Table 1:** Sample composition of empty liposomes (Lipo) and RIF-loaded liposomes (RIF–Lipo).

Sample	DPPG (mg/mL)	HSPC (mg/mL)	RIF (mg/mL)
Lipo	5	5	0
RIF–Lipo	5	5	5

Errors are within 5%.

**Table 2:** Physicochemical features of liposomal formulations.

Sample	Hydrodynamic Diameter ± SD (nm)	PDI ± SD	ζ-Potential ± SD (mV)	RIF *EE*%	Anisotropy (r) ± SD
Lipo	127.6 ± 0.8	0.20 ± 0.01	−55.4 ± 1.6	-	0.39 ± 0.03
RIF–Lipo	116.7 ± 0.9	0.20 ± 0.01	−41.7 ± 2.0	96 ± 10	0.34 ± 0.02

SD represents the standard deviation of data.

## Data Availability

The data presented in this study are available on request from the corresponding author.

## Supplementary Materials

The following are available online at https://www.mdpi.com/article/10.3390/pharmaceutics13071070/s1. Figure S1: Evaluation of direct mycobactericidal effect of rifampicin.
