# Supplementary material for: Rifampicin–Liposomes for Mycobacterium abscessus Infection Treatment: Intracellular Uptake and Antibacterial Activity Evaluation
Source: Pharmaceutics. 2021 Jul 13;13(7):1070. doi: 10.3390/pharmaceutics13071070 (PMC8309174; doi:10.3390/pharmaceutics13071070)
Supplement: Supplementary file 1 [file pharmaceutics-13-01070-s001.zip › pharmaceutics-1262158-supplementary.pdf]

pharmaceutics

Supplementary Materials: Rifampicin-liposomes for *Mycobacterium abscessus* infection treatment: intracellular uptake and antibacterial activity evaluation.

Federica Rinaldi, Patrizia Nadia Hanieh, Simona Sennato, Federica De Santis, Jacopo Forte, Maurizio Fraziano, Stefano Casciardi, Carlotta Marianecci, Federico Bordi and Maria Carafa

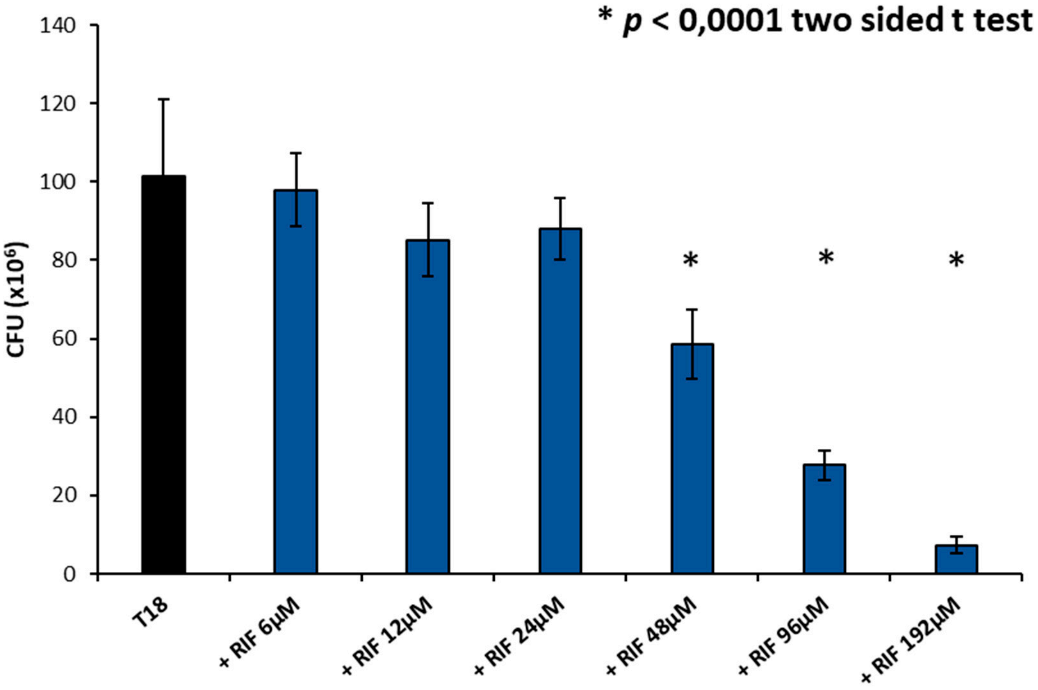

Figure S1. Evaluation of direct mycobactericidal effect of rifampicin.

Bacteria were cultured with different concentrations of Rifampicin (RIF) for 18 hours. Bacterial growth was quantified by CFU assay. Results are shown as mean ± standard deviation (SD) of CFU values performed in triplicate and are representative of three independent experiments. *$p<0.0001$ by two sided Student's t test in comparison with untreated control (T18).
